# Effect of Transcranial Direct Current Stimulation on Lower Extremity Muscle Strength, Quality of Life, and Functional Recovery in Individuals With Incomplete Spinal Cord Injury: A Randomized Controlled Study

**DOI:** 10.7759/cureus.51989

**Published:** 2024-01-10

**Authors:** Megha Nijhawan, Chitra Kataria

**Affiliations:** 1 Physiotherapy, Indian Spinal Injuries Centre (ISIC) Institute of Rehabilitation Sciences, Indian Spinal Injuries Centre, New Delhi, IND

**Keywords:** non-invasive brain stimulation, lower extremity motor scores, motor capabilities, rehabilitation, transcranial direct current stimulation, spinal cord injury

## Abstract

Background and objectives

Muscle strength and function are essential facets of rehabilitation for incomplete spinal cord injury (iSCI) patients. Various methods are being used to improve these outcome measures, but no gold standard method exists. Transcranial direct current stimulation (tDCS) is a relatively inexpensive, portable, readily available, and easy-to-use modality. It has shown promising results in many psychiatric and neurological conditions like stroke, cerebral palsy, and depression, but its role in spinal cord injury (SCI) is relatively unexplored. The study's objectives are to investigate the effect of anodal tDCS on lower limb muscle strength, quality of Life (QoL), and function in individuals with iSCI.

Methods

A randomized single-blinded sham control parallel-group study was conducted at the Indian Spinal Injuries Centre in New Delhi, India. There were 32 iSCI participants (28 males and four females) with 23 traumatic and nine non-traumatic etiologies. Participants were randomly assigned to receive 40 minutes of 2 mA anodal or sham stimulation over the targeted motor cortex areas for five sessions per week over two weeks.

The following outcome measures were measured at baseline after one and two weeks of the intervention: Lower Extremity Motor Score (LEMS), Spinal Cord Independence Measure (SCIM III), and WHO Quality of Life Bref (WHO QoL Bref).

Results

There was no significant difference at one week and two weeks of intervention for LEMS (p = 0.675, p = 0.978), SCIM III (p = 0.170, p = 0.133), WHO QoL Bref Domain 1 (p = 0.376, p = 0.282), Domain 2 (p = 0.728, p = 0.450), Domain 3 (p = 0.641, p = 0.993), Domain 4 (p = 0.294, p = 0.422), overall perception of QoL (p = 0.492, p = 1.000), and overall perception of their health (p = 0.300, p = 0.854) in the anodal and sham tDCS groups.

Conclusion

These primary findings suggest that anodal tDCS is ineffective in improving the QoL and motor and functional capabilities of individuals with iSCI. Further studies are necessary to determine whether it can be effective as a long-term rehabilitation strategy for the abovementioned population.

## Introduction

Spinal cord injury (SCI) results from an insult inflicted upon the spinal cord that affects its functions entirely or incompletely. It poses physical but also bio-psychological and socioeconomic consequences for the individual. It is a devastating condition that burdens society and the healthcare system [1]. Nearly half of the individuals with traumatic SCI suffer from an incomplete lesion, as per the epidemiological studies [2]. Due to the severance of neuronal continuity from the brain to the spine, patients exhibit impairments at various levels, with motor impairment being one of the primary concerns. It severely limits the patient's functional capacity and level of independence and may lead to secondary health problems [3]. It has also been observed that there is structural and functional reorganization at cortical and subcortical levels. The representation of the structures below the level of injury in the cortex is reduced post-SCI. The spinal cord and cortex show atrophic changes and reduced axonal integrity [4].

There is an extensive range of treatment strategies for individuals with SCI, including surgery, medication, physical therapy, occupational therapy, etc. Traditionally, the physical therapy treatment after SCI is limited to strengthening and stretching exercises, bed mobility exercises, neuromuscular stimulation, functional training, and so on [3]. There is a need to work on more rigorous treatment options beyond the traditional approach for motor recovery after SCI. In light of the role of enhancing residual corticospinal circuitry and primary motor cortex function [4], nowadays, there is a paradigm shift toward facilitating neuromodulation. There are various invasive and non-invasive means of facilitating neuromodulation. Non-invasive brain stimulation refers to techniques that act on brain physiology without internal electrode implantation through surgical means [5]. These techniques can potentially influence intracortical neuronal circuits and corticospinal tract excitability by altering neuronal transmembrane electric potentials [4].

Transcranial direct current stimulation (tDCS) is a non-invasive method of facilitating neuromodulation that has recently gained popularity because it is portable, inexpensive, and easy to use [6]. tDCS has been proposed as a tool that helps enhance neural plasticity and also helps the brain reach a favorable state of excitability, accelerating motor learning and training effects [3,4,6]. Though the mechanism of action is not completely clear to date, it has been postulated that supraspinal modulation by tDCS helps induce long-lasting spinal plasticity [7]. It has been used in normal subjects as well as in a variety of psychiatric and neurological conditions like stroke, cerebral palsy, depression, etc., for research and clinical purposes [5-6,8]. Though tDCS has shown promising results in the aforementioned conditions, a limited number of studies have explored its effect on SCI [6,8,9]. Among the few studies examining the impact of anodal tDCS on motor capabilities, most of the literature is on upper extremities, and there is hardly any literature on lower extremity motor strength [10,11].

As there is a dearth of literature about the effect of tDCS on lower extremity motor capabilities, the present study aims to analyze the effects of tDCS on motor and functional abilities in the lower extremities and quality of life (QoL) in the population mentioned above. The results of the present study will add to the literature on the role of tDCS in incomplete spinal cord injury (iSCI), thus optimizing treatment protocols and maximizing the effectiveness of tDCS in iSCI.

## Materials and methods

Study overview

This study investigates the effect of the anodal tDCS on Lower Extremity Motor Score (LEMS), function, and QoL in individuals with iSCI. A randomized, single-blinded, sham-control parallel-group study with 32 patients was undertaken at the Indian Spinal Injuries Centre in New Delhi, India. Patients with iSCI who met the eligibility criteria were recruited from the center's inpatient and outpatient rehabilitation departments. Participants were randomized to receive either anodal or sham tDCS. Each participant received 10 sessions of anodal or sham tDCS over two weeks. 

Ethical considerations

Oral and written information regarding the study's objectives, possible risks, and advantages of participation, as well as the option to withdraw before the screening, was provided to all prospective trial participants. Those interested in participating in the study were asked to sign a written informed consent form. The Indian Spinal Injuries Centre Institutional Ethics Committee has approved the study protocol (ISIC/RP/2019/017), and it is registered with the Clinical Trials Registry-India (CTRI/2020/02/023375). Data was collected, managed, and stored, and confidentiality was maintained during the collection and storage of the data. 

Study criteria

The eligibility criteria for the recruitment in the study are given in Table 1.

**Table 1:** Eligibility criteria AIS: ASIA impairment scale, SCI: spinal cord injury

Inclusion Criteria	Exclusion Criteria
Traumatic or non-traumatic cervical spinal cord injury	Any other pre-existing neurological problem, musculoskeletal condition, or psychiatric problem that may interfere with the treatment
AIS B&C&D	Spasticity of grade three or more on the Modified Ashworth Scale
Age: 18–60 Years	History of seizures or brain surgery
Gender: both male and female	Metallic brain implant
At least three months after SCI	Cardiac Pacemaker
Willing to participate in the study	Pregnancy

Study procedure

A patient information sheet was provided to all the participants, and the investigator obtained written informed consent before recruitment for the study. The investigator obtained the demographic details and recruited the participants in the study based on eligibility criteria after the examination. Before enrolling in the intervention, each participant had to obtain medical clearance from their treating physician.

The participants were assigned to anodal or sham groups randomly with the help of a computer-generated randomization sequence. Blinding was done for the patient's group allocation. However, because of the nature of the interventions, the principal investigator was notified of the group allocation. The treatment group remained unknown to the investigators conducting the data analysis.

**Figure 1:**
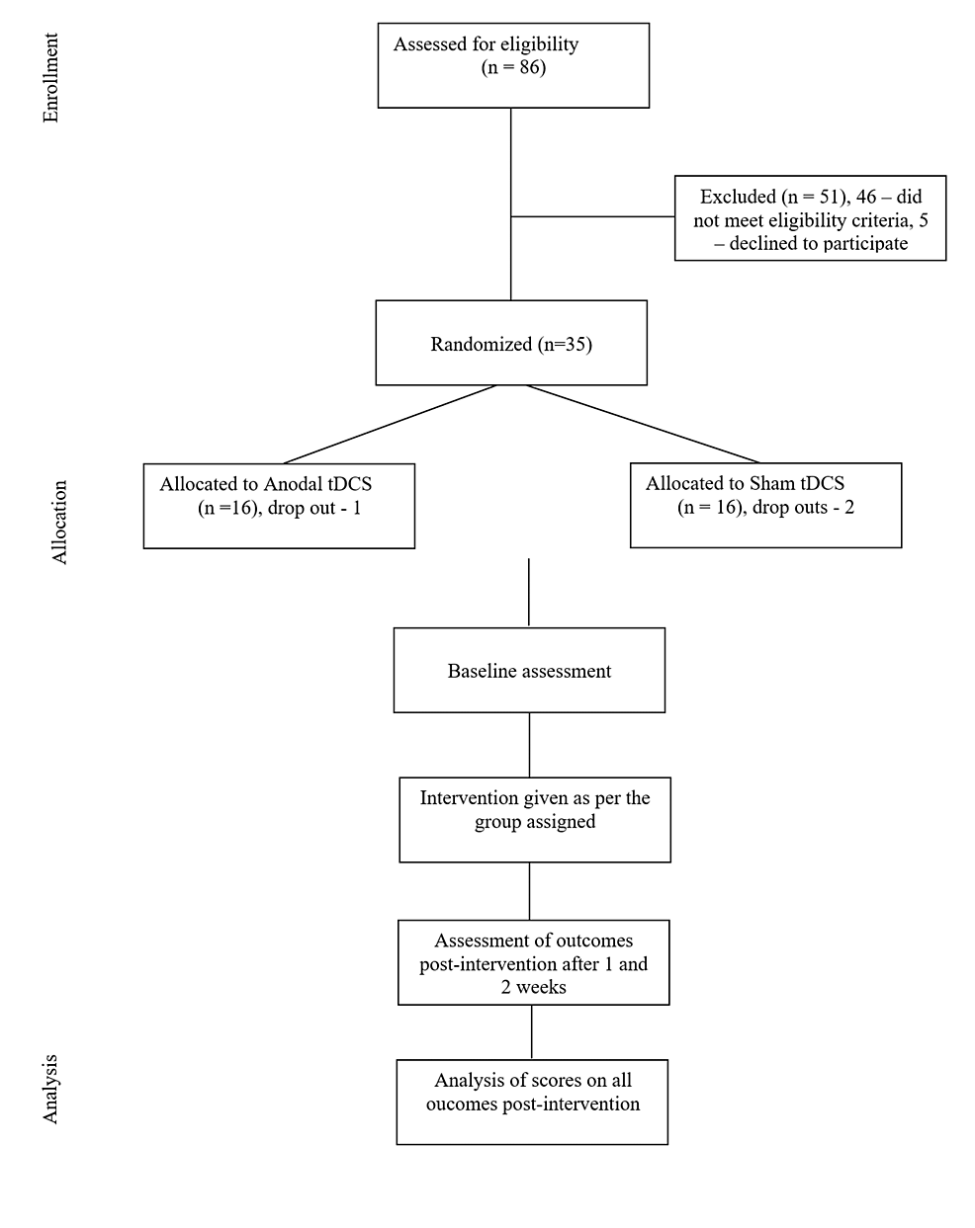
Consolidated Standards of Reporting Trials (CONSORT) flowchart. tDCS: Transcranial direct current stimulation

Table 2 describes the study enrollment, assessments performed, and interventions given. Baseline assessments were taken before the intervention. Participants received treatment as per the group assigned for two weeks with five sessions per week. The outcome measures were measured after one week and two weeks of the intervention to compare the short-term effects of the intervention. Our study design followed the Consolidated Standards of Reporting Trials (CONSORT) guidelines (Figure 1).

**Table 2:** Enrollment, interventions, and assessments schedule as per the Standard Protocol Items: Recommendations for Interventional Trials guideline. tDCS: Transcranial direct current stimulation; SCIM III: spinal cord injury index III; LEMS: lower extremity motor score; WHO QoL Bref: World Health Organization Quality of Life Bref; X-done

Variable	Enrollment (-t1)	Allocation	Baseline (T0)	-	-	
-	-	-	-	Intervention	Post-intervention assessment at week 1 (T1)	Post-intervention assessment at week 2 (T2)
Enrollment	-	-	-	-	-	-
Eligibility screen	X	-	-	-	-	-
Informed consent	X	-	-	-	-	-
Medical clearance	X	-	-	-	-	-
Allocation	-	X	-	-	-	-
Interventions	-	-	-	-	-	-
Anodal tDCS	-	-	-	X	-	-
Sham tDCS	-	-	-	X	-	-
Assessments	-	-	-	-	-	-
LEMS	-	-	X	-	X	X
SCIM III	-	-	X	-	X	X
WHO QoL Bref	-	-	X	-	X	X

The intervention included direct current via two saline-soaked sponge electrodes (1.5 inches in diameter) placed over intact skin on the scalp and delivered by a battery-driven tDCS device. Hair was spread to obtain maximum contact between the scalp and the electrode. The anode was placed over the primary motor area of the cerebral cortex according to the 10-20 international electroencephalogram (EEG) system (C3/C4) opposite to the target lower limb, and the cathode was placed over the supraorbital area on the opposite side of the active electrode [12-14]. The left and right lower extremity motor areas were stimulated for 20 minutes each. Five sessions per week over two weeks were given, i.e., 10 sessions. During tDCS, participants in the active group received 2 mA direct current throughout the session.

In contrast, in the sham group, the first one-minute participant received the stimulating current as the active group. The current intensity remained zero for the rest of the session [4,10-12]. All participants continued to receive usual care, which involved comprehensive rehabilitation. The participant's request for or reporting any adverse event was the criterion for discontinuation of the intervention.

Assessments

The same examiner made the assessments of all outcome measures before the intervention as a baseline reading and after one and two weeks of intervention. The variables assessed were LEMS, SCIM III, and WHO QoL Bref. These were assessed in a random order.

LEMS is the sum of the manual muscle testing (MMT) scores of five key muscle groups (hip flexors, knee extensors, ankle dorsiflexors, ankle plantar flexors, and extensor hallucis longus) on the right and left lower extremities at a neurological level of L2-S1 given by the ASIA Impairment Scale (AIS). Each muscle is scored ranging from zero to five, where zero indicates total paralysis, and five means a full range of active motion against gravity and full resistance, with a total achievable score of 50. The minimal clinically important difference is 3.66 [15,16].

The SCIM III is an instrument designed especially for the population with SCI that evaluates a range of activities of daily living. It is divided into movement, respiratory and sphincter control, and self-care. The subscales are weighted as follows: mobility, scored 0-40; breathing and sphincter management, scored 0-40; and self-care, scored 0-20, yielding a total score out of 100. Patients who need fewer aids or less assistance to do basic daily living and life support activities score higher [17].

The WHO QoL Bref is a 26-item tool with two general health and overall quality of life items and four domains (physical, psychological, social, and environmental). Two questions are assessed separately - question one (Q 1) examines an individual's overall perception of QoL, and question two (Q 2) evaluates the individual's overall perception of their health. Respondents on a five-point Likert-response scale assess the degree, frequency, or assessment of the chosen QoL attributes over the preceding two weeks. Higher scores denote higher QoL [18]. The Hindi WHO QoL Bref was used for participants who had difficulty filling out the English WHO QoL Bref.

Sample size calculation

The sample size was determined using a statistical test of difference between two independent means considering an effect size of 1.5, 5% level of significance, and 90% power. The sample size calculated was 11 per group. However, the sample size increased to 16 per group, with 40% lost to follow-up. The total sample size taken was 32. Post power was 91.69%. G*power 3.1.9.4 software was used to calculate the sample size and post power.

Statistical analysis

IBM SPSS Statistics for Windows, Version 21 (Released 2012; IBM Corp., Armonk, New York, United States) was used for statistical analysis. Descriptive data was exposed as means or proportions along with standard deviations. The Shapiro-Wilk test was applied to check if the data was normally distributed. Continuous variables were summarized as the mean ± standard deviation. A repeated measure, ANOVA, was used for within-group analysis, and a t-test was used for between-group analysis.

Statistical significance is set at p < 0.05. A p-value >0.05 is considered a non-significant difference, while a p-value < 0.05 is a significant difference. The value of the confidence interval is set at 95%.

## Results

A total of 32 individuals with iSCI participated in the study, 16 in each active and sham tDCS group. The mean age of participants in the active group was 30.0 ±10.60 years and 38.38 ± 10.12 years in the sham group (p = 0.618). In the anodal group of the 16 participants, two were classified as AIS B, nine were AIS C, and five were AIS D. In the sham group of the 16 participants, one was classified as AIS B, eight were AIS C, and seven were AIS D. The mean time since injury was 21.88 ± 20.24 for the anodal group and 17.88 ± 26.94 for the sham group. The groups did not differ significantly in age, time since injury, or mode of injury. Table 3 summarizes the demographic and clinical characteristics of the participants. All the participants tolerated tDCS well, and no adverse effects related to the application of tDCS were demonstrated.

**Table 3:** Baseline participants’ characteristics. AIS: ASIA Impairment Scale; tDCS: transcranial direct current stimulation; continuous data is presented as Mean ± SD; categorical data is presented as number (%); p< 0.05; *: statistically significant.

Participants’ characteristics		Anodal tDCS	Sham tDCS	p-value
N		16	16	-
Age (Years) Mean ± SD		30.00 ± 10.60	38.38± 10.12	0.618
Gender n (%)	Male	15 (93.8)	13 (56.3)	0.037*
	Female	1 (6.3)	3 (43.8)	
AIS n (%)	B	2 (12.5)	1 (6.3)	0.696
	C	9 (56.3)	8 (50.0)	
	D	5 (31.3)	7 (43.8)	
Etiology n (%)	Traumatic	12 (75.0)	11 (68.8)	0.694
	Non - traumatic	04 (25.0)	5 (31.3)	
Time since injury (months) Mean ± SD		21.88 ± 20.24	17.88± 26.94	0.752

At baseline, there was no significant difference between the groups for all the outcome variables: LEMS (p = 0.310), SCIM III (p = 0.211), WHO QoL Bref domain 1, 2, 3, 4, Q 1, and Q 2 (p = 0.887, p = 0.332, p = 0.806, p = 0.395, p = 0.191, and p = 1.000), as shown in Table 4.

**Table 4:** Between-group analysis at baseline. LEMS: Lower Extremity Motor Score; WHO QoL Bref: World Health Organization Quality of Life Bref; SCIM: Spinal Cord Injury Independence Measure; tDCS: transcranial direct current stimulation; Q 1: Question 1; Q 1: Question 2; data is presented as Mean ± SD; p< 0.05.

Outcomes		Anodal tDCS	Sham tDCS	p-value
LEMS (Mean ± SD)	-	23.69 ± 11.50	28.00 ± 11.88	0.310
WHO QoL Bref (Mean ± SD)	Domain 1	51.63 ± 14.67	50.88 ± 14.82	0.887
	Domain 2	48.50 ± 17.72	55.19 ± 20.52	0.332
	Domain 3	53.94 ± 17.86	53.06 ± 21.81	0.641
	Domain 4	53.75 ± 16.47	60.19 ± 17.59	0.294
	GQ 1	3.50 ± 0.894	3.69 ± 0.602	0.086
	GQ 2	3.38 ± 0.719	3.13 ± 0.619	0.166
SCIM III (Mean ± SD)	-	76.81 ± 11.50	71.69 ± 11.16	0.211

Between-group comparisons after one week of intervention did not reveal any significant difference in any outcome variable: LEMS (p = 0.675), SCIM III (p = 0.170), WHO QoL Bref domain 1, 2, 3, 4, Q 1, and Q 2 (p = 0.376, p = 0.728, p = 0.641, p = 0.294, p = 0.492, and p = 0.300), as shown in Table 5. Between-group comparisons after two weeks of intervention did not reveal any significant difference in any outcome variable: LEMS (p = 0.978), SCIM III (p = 0.133), WHO QoL Bref domain 1, 2, 3, 4, Q 1, and Q 2 (p = 0.282, p = 0.450, p = 0.993, p = 0.422, p = 1.000, p = 0.854), as shown in Table 5.

**Table 5:** Between-group analysis at one and two weeks of intervention. LEMS: Lower Extremity Motor Score; WHO QoL Bref: World Health Organization Quality of Life Bref; SCIM: Spinal Cord Injury Independence Measure; Q 1: Question 1; Q 2: Question 2; data is presented as Mean ± SD; p< 0.05.

Outcomes		Assessment time point	Anodal tDCS	Sham tDCS	p-value
LEMS (Mean ± SD)	-	One week	26.88 ± 12.99	28.75 ± 12.06	0.675
	-	Two weeks	30.00 ± 13.63	29.87 ± 11.50	0.978
WHO QoL Bref (Mean ± SD)	Domain 1	One week	56.75 ± 15.10	52.50 ± 11.42	0.376
	Domain 1	Two weeks	60.75 ± 13.59	54.81 ± 16.90	0.282
	Domain 2	One week	57.44 ± 18.79	55.19 ± 17.46	0.728
	Domain 2	Two weeks	61.81 ± 18.27	56.75 ± 19.15	0.450
	Domain 3	One week	56.25 ± 15.99	53.06 ± 21.81	0.641
	Domain 3	Two weeks	57.44 ± 16.66	57.38 ± 24.29	0.993
	Domain 4	One week	53.75 ± 16.47	60.19 ± 17.59	0.294
	Domain 4	Two weeks	57.13 ± 16.45	61.81 ± 16.15	0.422
	Q 1	One week	3.50 ± 0.894	3.69 ± 0.602	0.086
	Q 1	Two weeks	3.81 ± 0.655	3.81 ± 0.981	0.169
	Q 2	One week	3.38 ± 0.719	3.13 ± 0.619	0.166
	Q 2	Two weeks	3.38 ± 0.957	3.31 ± 0.946	0.714
SCIM III (Mean ± SD)	-	One week	77.38 ± 11.08	71.81 ± 11.28	0.170
	-	Two weeks	78.38 ± 11.35	72.13 ± 11.54	0.133

Within-group changes in the anodal group from baseline to one week and two weeks of post-intervention (Table 6) indicated significant improvement of LEMS after one week (p <0.001) and two weeks (p <0.001) of intervention; WHO QoL Bref domain two after one week (p = 0.015); WHO QoL Bref domain one, two, and Q 1 after two weeks (p = 0.005, p = 0.006, p = 0.046); and SCIM III after two weeks of intervention (p <0.001). 

**Table 6:** Within-group analysis – anodal group. LEMS: Lower Extremity Motor Score; WHO QoL Bref: World Health Organization Quality of Life Bref; SCIM: Spinal Cord Injury Independence Measure; Q 1: Question 1; Q 2: Question 2; data is presented as Mean ± SD; p< 0.05; *: statistically significant.

Outcomes		Pre	Post 1	Post 2	Post hoc (p-value)		
					Pre vs. Post 1	Pre vs. Post 2	Post 1 vs. Post 2
LEMS (Mean ± SD)		23.69 ± 11.50	26.88 ± 12.99	30.00 ± 13.63	0.000*	0.000*	0.000*
WHO QoL Bref (Mean ± SD)	Domain 1	51.63 ± 14.67	56.75 ± 15.10	60.75 ± 13.59	0.126	0.005*	0.019*
	Domain 2	48.50 ± 17.72	57.44 ± 18.79	61.81 ± 18.27	0.015*	0.006*	0.312
	Domain 3	53.94 ± 17.86	56.25 ± 15.99	57.44 ± 16.66	1.000	0.781	1.000
	Domain 4	53.75 ± 16.47	53.75 ± 16.47	57.13 ± 16.45	1.000	0.285	0.168
	Q 1	3.50 ± 0.894	3.50 ± 0.894	3.81 ± 0.655	0.564	0.046*	0.166
	Q 2	3.38 ± 0.719	3.38 ± 0.719	3.38 ± 0.957	0.332	0.696	1.000
SCIM III (Mean ± SD)		76.81 ± 11.50	77.38 ± 11.08	78.38 ± 11.35	0.210	0.000*	0.001*

Within-group changes in the sham group (Table 7) from baseline to one week and two weeks of post-intervention indicated significant improvement of LEMS after one week (p = 0.027) and two weeks (p <0.001) of intervention.

**Table 7:** Within-group analysis – Sham group. LEMS: Lower Extremity Motor Score; WHO QoL Bref: World Health Organization Quality of Life Bref; SCIM: Spinal Cord Injury Independence Measure; Q 1: Question 1; Q 2: Question 2; data is presented as Mean ± SD; p< 0.05; *: statistically significant.

Outcomes		Pre	Post 1	Post 2	Post hoc (p-value)		
					Pre vs. Post 1	Pre vs. Post 2	Post 1 vs. Post 2
LEMS (Mean ± SD)		28.00 ± 11.88	28.75 ± 12.06	29.87 ± 11.50	0.027*	0.000*	0.008*
WHO QoL Bref (Mean ± SD)	Domain 1	50.88 ± 14.82	52.50 ± 11.42	54.81 ± 16.90	1.000	0.597	0.932
	Domain 2	55.19 ± 20.52	55.19 ± 17.46	56.75 ± 19.15	1.000	1.000	1.000
	Domain 3	53.06 ± 21.81	53.06 ± 21.81	57.38 ± 24.29	1.000	1.000	0.475
	Domain 4	60.19 ± 17.59	60.19 ± 17.59	61.81 ± 16.15	0.215	0.468	1.000
	GQ 1	3.69 ± 0.602	3.69 ± 0.602	3.81 ± 0.981	1.000	1.000	1.000
	GQ 2	3.13 ± 0.619	3.13 ± 0.619	3.31 ± 0.946	1.000	0.711	1.000
SCIM III (Mean ± SD)		71.69 ± 11.16	71.81 ± 11.28	72.13 ± 11.54	0.492	0.206	0.166

## Discussion

Most studies published on tDCS in SCI pertain to the upper extremities. Research studies exploring the effects of tDCS on lower extremity motor learning are limited, mostly with a small sample size. They are combined with robot-assisted motor training or other mass practice [10,11]. Thus, this study aimed to explore the effectiveness of anodal tDCS and conventional physiotherapy on lower limb motor and functional capabilities in iSCI.

This study demonstrates that anodal tDCS and conventional rehabilitation can be safe, as no adverse event was reported during either anodal or sham tDCS. However, it is ineffective in improving motor and functional outcome measures in individuals with iSCI.

For LEMS, both groups improved significantly after one and two weeks of intervention, but they did not differ significantly from each other. Plausible causes of the anodal group not showing significant improvement may be: first, increased brain excitability may not translate into processing efficacy and clinical improvement, and vice versa [10,19].

Second, all the participants were taking medications for either pain or spasticity, which are known to affect the excitability of the cortical and spinal circuits. Baclofen and gabapentin, for instance, have been demonstrated to lower intracortical facilitation, thereby reducing excitability [20]. As a result, our findings may have been influenced by the various medications taken by each subject. Third, we used a 10-20 EEG system to identify C3/C4 and did not use magnetic resonance imaging-guided neuronavigation or motor-evoked potentials (MEP).

Our study's results align with the study by Kumru et al. in 2016, who did not observe any significant effect of anodal tDCS along with lokomat gait training over lokomat gait training alone in iSCI patients [10]. The dosage given was similar to the present study, with a 2 mA current for 20 minutes. However, the number of sessions was double that of the present study.

Raithatha et al. (2016) observed significant improvement in MMT of lower limb muscles in the anodal stimulation group [11]. They might have observed better motor outcomes in the anodal group due to the large number of sessions (36). Also, the electrode montage they used was different from the present study. They gave anodal stimulation to the vertex (Cz) instead of the primary motor area of the cerebral cortex (C3/C4). Their results must be interpreted cautiously, as the sample size was 15 [11].

A study by Khedr et al. (2013) showed a marginally significant improvement in muscle strength in the active tDCS group over the sham tDCS group in the ischaemic stroke population. Participants received six consecutive sessions of 25 minutes each [21]. Their observations may differ from the present study due to different phases of rehabilitation as all their participants were in the early stage of stroke rehabilitation (<30 days post-stroke), and participants in the present study had a variable duration of time post-injury.

On the other hand, like the results of the present study, Montenegro et al. (2016) observed no significant improvement in the motor capability of the quadriceps muscle (peak muscular torque) after a single 20-minute session of 2mA tDCS in individuals with stroke over healthy individuals. However, only one session was given in the stroke population compared to 10 in the present study [22]. The results are also mixed in the Stroke population due to differences in methodology over montage, current intensity, duration of stimulation, number of sessions, and differences over the study population regarding type and chronicity of Stroke. 

For SCIM III, although the anodal group showed significant improvement after two weeks of intervention on within-group analysis, there was no significant difference between the groups. Raithatha et al. (2016) observed similar results when they explored the effect of anodal stimulation and robot-assisted gait training in individuals with iSCI [11]. In a study on chronic stroke by Danzl et al. (2013), no significant improvement in function (Stroke Impact Scale 16) was seen in the anodal tDCS group over the sham tDCS group after 12 sessions. However, this study had a small sample of four participants in each group [23]. 

QoL showed no significant improvement in our study's anodal group over the sham group. However, within-group analysis shows significant improvement in the anodal group after two weeks of intervention for the psychological domain and the individual's perception of the overall QoL. Furthermore, the physical domain improved significantly after one week and two weeks of intervention in the anodal group. If the intervention period was longer, the anodal group may have significantly improved over the sham group. Previously done studies on the effectiveness of tDCS in individuals with iSCI have not taken QoL as an outcome measure, so we do not have any previous literature to compare with.

However, our results align with a study by Khandare et al. (2020). They explored the effects of three weeks of tDCS intervention on the QoL in individuals with stroke. They found no significant improvement in the active group over the sham stimulation group [24].

We did not observe any statistically significant improvement in the active tDCS group, which may be because we gave only 10 sessions, and our participants were heterogeneous in terms of etiology, the severity of SCI, time since injury, and neurologic level of injury. Our study being a single-blinded trial with just the patient blinded is one of its limitations. Additionally, we took a convenience sample from a single rehabilitation facility in New Delhi to select the participants. The participant recruitment from a single center could have led to a potential bias in selection in our study.

Clinical implication

The role of tDCS is evolving in the management of SCI. Very few studies address the effect of tDCS on motor strength variables iSCI, and even less in the lower extremities. The dosage, electrode size, electrode montage, and methods used in different studies to find the stimulation areas are variable.

Each well-designed study adds vital information to the effects of tDCS in managing individuals with iSCI. The results of the present study will contribute to the design of a better tDCS protocol for the rehabilitation of individuals with iSCI.

## Conclusions

In conclusion, an anodal tDCS protocol of 10 sessions over two weeks with a 2 mA current over the C3/C4 motor area (10-20 EEG) for 20 minutes each is not more effective than sham tDCS for improving LEMS, QoL, and function in persons with iSCI.

Further studies performed in a more homogeneous population regarding the impairment level (AIS) and time-lapse since the injury with a larger population are needed to verify the potential clinical impact of our protocol of non-invasive brain stimulation in iSCI patients. Follow-up assessments will explore whether participants who received the active tDCS protocol can retain their improvements for longer than participants who did not.

It is possible that tDCS is more effective when combined with other non-invasive electrical stimulation therapies. Future studies shall explore the combined effect of tDCS and transcutaneous spinal direct current stimulation or patterned electrical stimulation in SCI management. Further studies are recommended to quantify the neuroplastic effects of anodal tDCS with objective measures of neuroplasticity like functional magnetic resonance imaging, EEG/evoked potentials, and transcranial magnetic stimulation.
